# Opsonic function of sialic acid specific lectin in freshwater crab *Paratelphusa jacquemontii*

**DOI:** 10.1186/s40064-015-1349-0

**Published:** 2015-10-13

**Authors:** Maghil Denis, Karthigayani Thayappan, Sivakumar Mullivanam Ramasamy, Arumugam Munusamy

**Affiliations:** Department of Zoology, University of Madras, Guindy Campus, Chennai, 600025 India

**Keywords:** Sialic acid, Lectin, Erythrocyte, Phagocytosis, Opsonin, Hemolysis

## Abstract

The sialic acid specific humoral lectin, Pjlec of the freshwater crab *Paratelphusa jacquemontii* was investigated for its opsonin function with rabbit erythrocyte as target cell for phagocytosis by the crab’s hemocyte. The untreated or trypsin treated erythrocyte induced lectin response after challenge however failed when treated with neuraminidase evidently indicating glycan dependency for elicited immune response. Our observation of in vitro phagocytosis of the erythrocyte untreated or coated with serum, clarified serum appeared to be recognized and engulfed by hemocytes but when coated with isolated lectin Pjlec, the response was elicited. Moreover, with trypsin treated erythrocyte the response remained unchanged but neuraminidase or *O*-glycosidase treatment eliminated the response reaction. This suggested the sialic acid specific reaction of lectin with the erythrocyte and was essential for recognition to allow the lectin Pjlec to act as an opsonin. The flowcytometry observation affirmed the enhancement of phagocytosis by Pjlec coated hemocyte. The efficiency of in vitro hemolysis of Pjlec coated erythrocyte with hemocyte when compared to untreated erythrocyte with or without hemocyte also established the opsonic function of the lectin. The mechanism of phagocytosis and induction were dependent on specific recognition of the erythrocyte by the multivalent binding site of the lectin protein, and the elicitation of the immune response was a function of the sialoglycan surface. The pathway of the challenge suggested that after entry of nonself recognition by lectin was followed by induction and activation of phagocytosis by opsonic binding of the lectin.

## Background

The innate immune system in crustacea although primitive has evolved a complex and efficient form, comprising of humoral and cellular components to recognize the pathogens by pathogen associated molecular patterns (PAMPs) through pattern recognition receptors (PRR) that activate conserved host defense signaling pathways to trigger or control the expression of a variety of immune responsive genes (Akira et al. 2006; Medzhitov and Janeway 2002). Among the PRR in crustacea, lectins have been reported to recognize and act as opsonin in phagocytic responses (Weinheimer et al. 1970; Ofek and Sharon 1988; Sahly et al. 2008). Lectins are multivalent proteins or glycoproteins with binding specificity to monosaccharides or oligosaccharides (Lis and Sharon 1986). that enables specific interaction with glycans on cells or pathogens for recognition and discrimination of self from non self (Baronde 1981). The mechanism of phagocytosis consists of multistep processes and is complex varying among species based on the differences in functioning of recognition, binding and ingestion. It has been a matter of speculation if the hemocyte receptors are able to recognize the non self independent to opsonin or are dependent on opsonin for recognition and subsequent internalization of foreign entity. The carcinolectins 5, CL5a and CL5b in the horseshoe crab *Carcinoscorpius rotundicauda*, are the major proteins that bind to many pathogens and initiate the activation of a complement-like system, facilitating the phagocytosis of pathogens (Zhu et al. 2006). The C-type lectin, FcLec4, from the Chinese white shrimp *Fenneropenaeus chinensis* was reported to interact with the pathogenic bacterium *Vibrio anguillarum* and facilitate bacterial clearance in vivo and functioned as an opsonin by protein interaction with β-integrin on hemocyte surface during clearance (Wang et al. 2009). A galectin from the kuruma shrimp *Marsupenaeus japonicas* functions as an opsonin for microbial pathogens, interacting with hemocyte surface promoting their phagocytosis (Wang et al. 2014).

Lectin which are defined in its function by its sugar specificity bind and recognize sialoconjugates on cell surface (Marques and Barracco 2000). Sialic acid constitutes a family of nine carbon sugars, neuraminic acid with diverse structural forms and occurs at the terminal glycan of the cells and microbes. Crustaceans lack the ability to synthesize sialic acid (Warren 1963; Segler et al. 1978) however lectins with difference in ligand affinity to the various structural forms of neuraminic acid (NeuAc) have been identified and isolated in lobsters, crabs and prawns (Vazquez et al. 1993; Ravindranath and Cooper 1984; Mercy and Ravindranath 1993). Among the variants of NeuAc, the *O*-acetylated sialic acidmediate cell–cell interactions in organogenesis, immune regulation, and pathophysiological processess (Kelm and Schauer 1997; Schauer et al. 1995; Varki 1992). In the present study the *O*-acetyl neuraminic acid (*O*-NeuAc) specific lectin Pjlec isolated from the hemolymph of the freshwater crab *Paratelphusa jacquemontii* (Denis et al. 2003) was used to investigate the cellular interactions of circulating hemocytes as phagocyte host cell with rabbit erythrocyte as target cell. Earlier reports have demonstrated that the outer membrane protein (OMP) of microbial origin that contain LPS, beta glucan and proteins are effective immunostimulants in enhancing innate immune response in shrimp and also non-specific and specific defense systems of fish (Maftuch et al. 2013) including macrophage phagocytic activity (Jin et al. 2008). The present study demonstrates the recognition process of nonself by Pjlec lectin based on specific ligand binding to surface glycan and the subsequently regulate the process of phagocytosis.

## Methods

### Maintenance of animals

The fresh water crab *Paratelphusa jacquemontii* was collected from the paddy fields in Kanyakumari district, Tamil Nadu, India and maintained in tanks (45 cm × 75 cm) immersed in water and being a tropical place the temperature remained moderate (27–30 °C) throughout the year. The crabs were fed with paddy or fish meat daily.

### Hemolymph extraction

Hemolymph was collected from uninjured non-autotomised and intermoult male or female crabs. For larger crabs, after cutting the dactylus, the hemolymph was allowed to bleed and pooled in centrifuge tubes placed on ice and for smaller crabs the hemolymph was extracted using a sterile 1.0 ml syringe and 22 gauge needle from arthroidal membrane at the base of chelipeds and walking legs.

### Serum preparation

The pooled hemolymph was allowed to clot on ice for 1-4 h and then centrifuged (5000×*g* 4 °C), to collect the serum, that could be utilized immediately or dated and stored in a freezer to be used within a month.

### Clarified serum preparation

Clarified serum was obtained by sedimentation of hemocyanin by centrifugation at 15 × 10^4^×*g* for 2 h at 4 °C (Beckman T 65 rotor).

### Erythrocyte preparation

Rabbit blood was used for hemagglutination assay and was obtained from the vein in the ear using syringe with 22 gauge needle. Erythrocytes were collected directly in modified alsever’s medium, (Sodium Citrate 30 mM, NaCl 77 mM, Glucose 114 mM, Neomycin Sulfate 100 μg/ml, Chloramphenicol 330 μg/ml, pH 6.1). Before use the rabbit erythrocyte was washed thrice in saline (0.9 % NaCl) by centrifugation (1500 rpm, 5 min at RT) and finally 1.5 % erythrocyte suspension was prepared in Tris-buffer (Tris–HCl 50 mM, NaCl 100 mM, and CaCl_2_ 10 mM, pH 7.5.

### Protein determination

Protein concentration was determined following the method of (Bradford 1976) using bovine serum albumin as the standard.

### Isolation of lectin by BSM-activated Sepharose affinity chromatography

The lectin Pjlec was isolated from the hemolymph of the crab by affinity chromatography using bovine submaxillary mucin, BSM-activated Sepharose 4B as described in the earlier report (Denis et al. 2003). The purity and activity of Pjlec was confirmed by hemagglutination activity and sugar specificity of the lectin (HAI) before experimenting for coating rabbit erythrocyte.

### Enzyme treatment of rabbit erythrocyte

*Trypsin treatment* The rabbit erythrocyte washed in Tris–HCl buffer (Tris 50 mM, NaCl 100 mM, and CaCl_2_ 10 mM, pH 7.5) were prepared as 1.5 % suspension in the same buffer, mixed with 1:1 v/v of trypsin (1 mg/ml.) were incubated at 37 °C for 1 h and washed in TBS prior to hemagglutination. For in vitro phagocytosis assay and in vivo induction of lectin, phosphate buffered saline (PBS) pH 7.0 was used to wash and prepare the 10 % suspension of trypsin treated rabbit erythrocyte.

Neuraminidase Treatment: A reaction mixture (total 5 ml) containing 10 % washed rabbit erythrocytes in PBS (0.01 M sodium phosphate, pH 6.9 and 0.145 M NaCl) and 140 mU ml/l of neuraminidase of *Clostridium perfringens* (Type X: Sigma) was incubated at 37 °C for 4 h. The treated cells were washed with PBS three times and pelleted by low speed centrifugation. Finally they were washed and suspended in PBS was used for the experiment on in vivo induction and in vitro phagocytosis assays.

The bundle of neuraminidase and *O*-glycosidase (NEB, England) treatment of erythrocyte was followed as given in the instructions and prepared as given for neuraminidase for in vivo induction and in vitro phagocytosis assays.

### Induction of hemagglutination

The rabbit erythrocyte suspended in phosphate-buffered saline PBS (0.01 M sodium phosphate, pH 6.9 and 0.145 M NaCl) of 10 % concentration (0.1 ml) was administered into the soft arthroidal membrane between the coxa of the fourth pleopod and the dorsal surface of the carapace in separate groups of crabs comprising 10 crabs each. The effect of administered erythrocyte was analyzed by HA assay in the serum from the hemolymph collected at time periods of 30 min, 1, 2, 4, 8, 16, 24, 48 and 72 h. The HA assays of untreated crabs were taken as control.

The lectin coated rabbit erythrocyte was prepared by incubating a suspension of erythrocyte (200 μl) in 20 volumes of lectin (diluted to sub-agglutinating concentration) for 1 h (RT). The lectin coated erythrocyte was washed and resuspended to 10 % sterilized PBS and used for challenge.

### Clearance of erythrocyte

To determine the clearance of erythrocyte administered into the crab from the circulation, hemolymph (100μl) was collected with a micropipette at regular time intervals (5 min) and diluted to 1 ml with double distilled water for estimation of hemoglobin (Tietz 1976) until the erythrocyte completely disappeared from the circulation. The rate of clearance before and after lectin coating was compared.

Resuspended 200 μl of washed and packed erythrocyte in 20 volumes of lectin (diluted to sub-agglutinating concentration) and incubated the mixture for 1 h at 30 °C. The lectin coated erythrocyte were washed and resuspended in sterilized PBS and were injected as stated earlier. The rate of clearance before and after lectin coating was compared.

### Hemolysis

Hemocytes in the hemolymph (150 µl) of the crab collected in 10 ml of pre-chilled iso-osmotic buffer (TBS: 50 mM Tris, 210 mM NaCl, 5 mM KCl, 2.5 mM MgCl_2_, 100 mg d-glucose, pH 7.5, 480 mOsm) were separated by centrifugation (2000×*g*, 2 min at 4 °C) and washed twice to remove the contaminating proteins. The hemocyte suspension (100 μl) in iso-osmotic buffer containing 1.0 × 10^6^ cells/ml was added to 100 μl of 1.5 % of the rabbit erythrocyte suspension in the same buffer including one batch of rabbit erythrocyte pretreated with Pjlec lectin at sub-agglutinating concentration (1:10 dilution). The untreated and Pjlec treated hemocytes with were incubated for 1 h at 30 °C, and then the erythrocyte-hemocyte mixture was centrifuged at 2000×*g*, 2 min at 4 °C. The supernatant was collected for estimation of hemoglobin content, following the method of (Tietz 1976). The control included lectin coated or uncoated erythrocyte without hemocytes. The hemolysis of erythrocyte was measured at intervals of 5 min and percentage of hemolysis estimated and graphically represented.

### Phagocytosis

#### Preparation of hemocyte monolayers

Hemolymph (100 µl) collected in 10 ml of iso-osmotic buffer (TBS: 50 mM Tris, 210 mM NaCl, 5 mM KCl, 2.5 mM MgCl_2_, 100 mg d-glucose, pH 7.5, 480 mOsm) was spread on an alcohol-washed, clean, dry glass slide over an area of 2 cm^2^ and kept in a moist chamber for 30 min at 23 °C to obtain hemocyte monolayer.

#### Viability of hemocytes

The viability of hemocytes in monolayers was determined up to 2 h using trypan blue dye exclusion technique following (Garvey et al. 1979). The percentage of hemocyte viability was calculated as follows:

$${\text{Percentage of viable cells}} = {\text{Number of live cells counted}}/{\text{Total number of cells counted }} \times { 1}00.$$

#### Preparation of erythrocyte for phagocytosis assay

Rabbit blood collected in Alsever’s solution, was fixed in glutaraldehyde following (Nowak et al. 1976).

#### Pretreatment of rabbit erythrocyte with serum, clarified serum and isolated lectin

Sub agglutinating concentration of each sample (10 ml), serum (92.35 mg ml^−1^ protein), clarified serum (2.3 mg ml^−1^ protein) and isolated lectin (0.04 mg.ml^−1^ protein) by diluting to titre value 2 with iso-osmotic TBS buffer, was mixed with 50 μl of washed and pelleted rabbit erythrocyte for 1 h at RT with gentle shaking. After incubation, the treated erythrocyte were washed by centrifugation (4000×*g*, 5 min at 4 °C) and resuspended in the iso-osmotic TBS as 1.5 % v/v suspension.

#### In vitro phagocytosis assays

Phagocytosis of rabbit erythrocyte in four hemocyte monolayers were prepared using hemolymph samples obtained from crabs, first and second pair of monolayers was overlaid with 200 μl rabbit erythrocyte and observed at 5 min interval for 1 h. The mean of five determinations were taken as control.

#### In vitro opsono phagocytosis of rabbit erythrocyte pre-treated with serum, clarified serum and isolated lectin Pjlec

The rabbit erythrocyte pretreated (coated) with serum, clarified serum and Pjlec lectin from *P. jacquemontii* served as target cell for in vitro opsono phagocytosis assay. The analysis was set separately for each sample of pretreated erythrocyte and for each set of experimental analysis four hemocyte monolayers were prepared using hemolymph obtained from the crab. The first pair of monolayer was overlaid with 200 μl of rabbit erythrocyte pre-treated with serum or clarified serum or isolated lectin at sub agglutination dilution, and the second pair of monolayer was overlaid with untreated rabbit erythrocyte which served as control, and each was suspended in iso-osmotic buffer.

#### In vitro phagocytosis of enzyme treated rabbit erythrocyte

In vitro phagocytosis analysis was set with trypsin or neuraminidase treated rabbit erythrocyte as target cell and the experiment was set as described for in vitro opsono phagocytosis assay.

#### Calculation

$${\text{Percentage phagocytosis}} = {\text{No}}.{\text{ of phagocytotic hemocytes}}/{\text{Total no}}.{\text{ of hemocytes }} \times 100.$$

### Flow cytometry

#### Hemocyte population

**H**emolymph (100 µL) extracted from the excised dactylus of the crab was collected in 900 µl of pre-chilled iso osmotic buffer (TBS: 50 mM Tris, 210 mM NaCl, 5 mM KCl, 2.5 mM MgCl_2_, 100 mg d-glucose, pH 7.5, 480 mOsm)in poly propylene tube was examined in flow cytometry (BDFAC5 JAZZ) by excitation with 480 nm to obtain Side scattered light (SSC) and Forward scattered light (FSC) computed to obtain the quantative assessment data representing the percentage of hemocyte morphotypes subpopulations in the cytogram.

#### Phagocytosis

FITC annexin V stained rabbit erythrocyte was incubated with the sample prepared by collecting fresh hemolymph (100 µl) in 900 µl of pre-chilled iso osmotic buffer in poly propylene tube, for 10 min and examined in flow cytometer to computate the data and obtain the dot plot of the fluorescent stained erythrocyte.

### Statistical analysis

The mean and standard deviation for the experiments was evaluated for the control and experimental. The difference between control and experimental values or between two experimental values was tested for statistical significance using ANOVA-single way classification following (Sokal and Rohlf 1973).

## Results

### Hemagglutination assay (HA)

As shown in Table 1, the hemagglutination activity (HA) of serum, clarified serum and purified lectin (Pjlec) against rabbit erythrocyte was determined and found to be around a median titer of 2048. The trypsin treated rabbit erythrocyte also showed no variation in HA and showed a value of 2048. However, HA of neuraminidase treated rabbit erythrocyte decreased to 4 and 2 in the tested samples (Table 1).

**Table 1:** Hemagglutination activity (HA) of serum of clarified and purified lectin (Pjlec) with untreated and enzyme treated rabbit erythrocyte in *Paratelphusa jacquemontii*

S. no	Sample	Protein (mg/ml)	Hemagglutination activity (HA)			
			Untreated rabbit erythrocyte	Enzyme treated rabbit erythrocyte		
				Trypsin^a^	Neuraminidase^b^	*O*-glycosidase^c^
1	Serum	17.78 ± 2.35	2048	2048	4	2
2	Clarified serum	2.35 ± 0.67	2048	2048	4	2
3	Purified lectin	0.27 ± 0.14	2048	2048	4	2

Data represents median titer value from 10 determinations of serum, clarified serum and isolated lectin Pjlec against untreated and enzyme treated rabbit erythrocyte

Each sample (25 µl) serially diluted in Tris buffer saline (Tris: 50 mM, NaCl 100 mM, CaCl_2_ 10 mM pH 7.5) and 1.5 % suspension of rabbit erythrocyte in same buffer was added and incubated for 1 h to observe hemagglutination titer

Control: untreated rabbit erythrocyte

Enzyme treated erythrocyte:

^a^Proteolytic enzyme -Trypsin (1 mg/ml) treated rabbit erythrocyte

^b^Asialo rabbit erythrocyte : Neuraminidase (0.1 unit of Clostridium perfringens sialidase (Type X), in PBS-BSA (0.01 M sodium phosphate, and 0.145 M NaCl) pH 7, 37 °C for 4 h)

^c^O-acetyl neuraminidase (O-glycosidase)

### Induction of agglutinin activity by rabbit erythrocyte

**Fig. 1:**
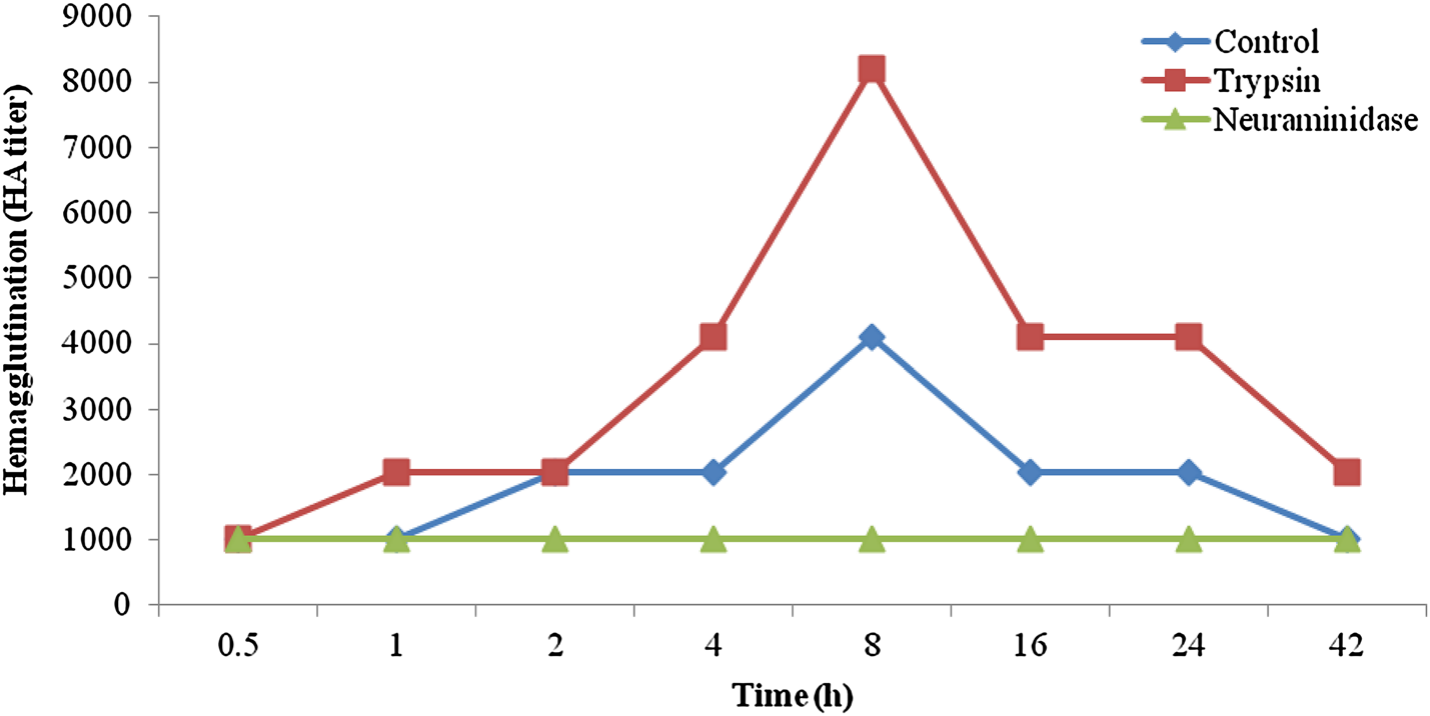
Induction of hemagglutination activity in the hemolymph agglutinin of *Paratelphusa jacquemontii* by administration of untreated, trypsin treated and asialo rabbit erythrocyte. *Graph* represents median HA titer values from 10 determinations with the administration of 0.1 ml untreated (control), trypsin (1 mg/ml) and neuraminidase 100 mU/100 µ enzyme treated 10 % suspension of rabbit erythrocyte in PBS (0.01 M sodium phosphate, pH 6.9 and 0.145 M NaCl)

The challenge of 10 % rabbit erythrocyte elicited lectin activity to HA titer value of 8192 between 4 and 16 h of its administration and a challenge of trypsin treated rabbit erythrocyte induced one fold increase in lectin activity to 4096 after 8 h of challenge. However, induction of lectin activity failed to occur with neuraminidase treated rabbit erythrocyte (Fig. 1).

### Hemocyte-mediated hemolysis

**Fig. 2:**
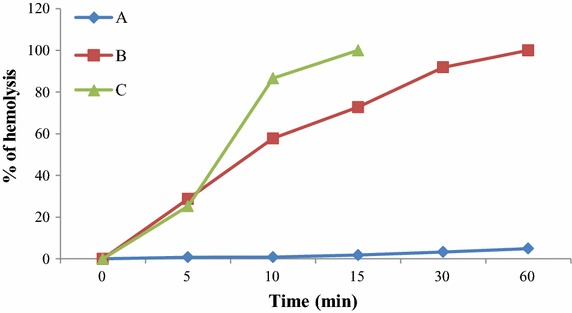
Hemolysis of untreated and Pjlec coated rabbit erythrocyte with or without hemocytes from the hemolymph of *Paratelphusa jacquemontii.* Hemolysis was determined from hemoglobin liberated at 5, 10, 15, 30 min and 1 h and percentage of hemolysis evaluated. Data represents average of five determinations. *A* Hemolysis % of Pjlec coated rabbit erythrocyte. *B* Hemolysis % of rabbit erythrocyte with hemocytes. *C* Hemolysis % of Pjlec coated rabbit erythrocyte with hemocytes

The percentage of hemolysis as recorded for hemocytes, lectin Pjlec and hemocytes with erythrocyte or lectin coated erythrocyte is represented graphically in Fig. 2. Lectin Pjlec or hemocytes had weak hemolytic effect on the erythrocyte. The lectin Pjlec coated erythrocyte with hemocytes facilitated hemolysis of the erythrocyte in comparison to the untreated erythrocytes with hemocytes (*p* < 0.05).

### Clearance of rabbit erythrocyte

The lectin-coated rabbit erythrocyte was cleared in less than 10 min whereas uncoated erythrocyte took 20 min for clearance and this variation was significant (*p* < 0.05).

### Phagocytosis

**Fig. 3:**
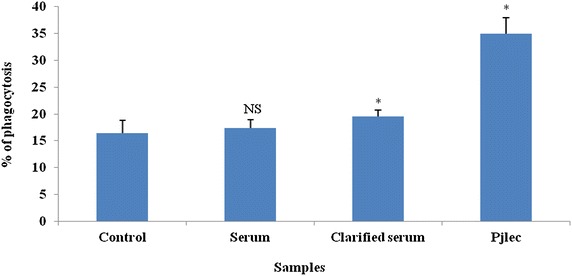
In vitro phagocytosis of untreated and serum, clarified serum and lectin Pjlec coated rabbit erythrocyte by hemocytes in the hemolymph of *Paratelphusa jacquemontii.* In vitro phagocytosis in percentage, % of rabbit erythrocyte suspended in iso-osmotic buffer, TBS: 50 mM Tris, 210 mM NaCl, 5 mM KCl, 2.5 mM MgCl2, 100 mg d-glucose, *p*H 7.5, 480 mOsm (control 16.48 %) or serum (92.35 mg ml^−1^) coated erythrocyte 17.54 %, clarified serum (2.3 mg ml^−1^) coated erythrocyte 19.50 % and lectin (0.04 mg ml^−1^) coated erythrocyte 35 % or anti lectin (3.5 mg ml^−1^) 17.55 %. *Vertical bars* represent mean ± SD of three determinations. *Asterisk* the variation by one way ANOVA in percentage of phagocytosis between control and clarified serum or lectin coated erythrocyte were statistically significant (*p* < 0.05). *NS* not significant

The monolayer of hemocytes prepared on a glass slide (23 °C) was overlaid with rabbit erythrocyte and observed under phase optics at 40× magnification. Initially within ten min the hemocytes appeared to show extensions of the cytoplasm in response to the target cell and in about 20–30 min the erythrocyte cells were observed to move and bind to hemocyte surface to be ultimately ingested in another 10–15 min. Of the total hemocytes evaluated of about 27.25 ± 10 the semigranular and granular cells were observed to show higher phagocytotic rate of 16.48 ± 2.31 % which increased to 17.54 ± 1.5 % with serum coated erythrocyte. However phagocytic rate increased with significant variance to 19.50 ± 2.43 % with clarified serum coated erythrocyte and to 35 ± 3.25 % with Pjlec lectin coated erythrocyte (*p* < 0.05) as shown in Fig. 3.

**Fig. 4:**
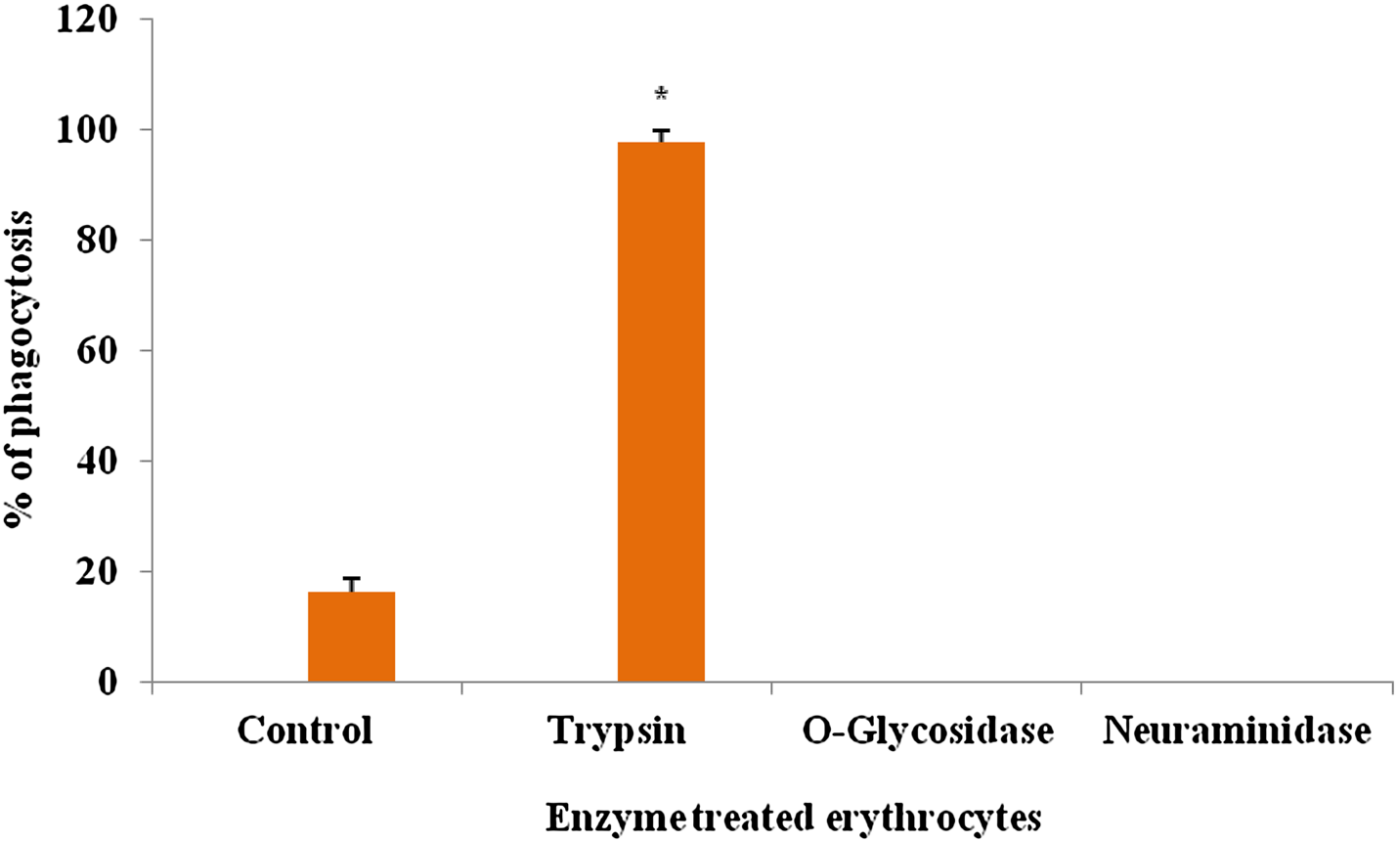
In vitro phagocytosis of untreated and enzyme treated rabbit erythrocyte by hemocytes in the hemolymph of *Paratelphusa jacquemontii.* Control: rabbit erythrocyte 1.5 %. Enzyme treated erythrocyte: trypsin: rabbit erythrocyte 1.5 % 500 µl treated with 10 µg of trypsin, O-glycosidase: rabbit erythrocyte 0.5 % 500 µl treated with 1 µl of O-Glycosidase (NEB), neuraminidase: rabbit erythrocyte 0.5 % 500 µl treated with 1 µl h 1 µl of Neuraminidase (Sigma). *Vertical bars* represent mean ± SD of three determinations. *Asterisk* the variation by one way ANOVA in % phagocytosis was significant (*p* < 0.05)

The trypsin treated erythrocyte remained the same as untreated erythrocyte (control) whereas the glycosidase and neuraminidase treated erythrocyte failed to display phagocytosis (*p* < 0.05) as observed in Fig. 4.

### Flow cytometry

**Fig. 5:**
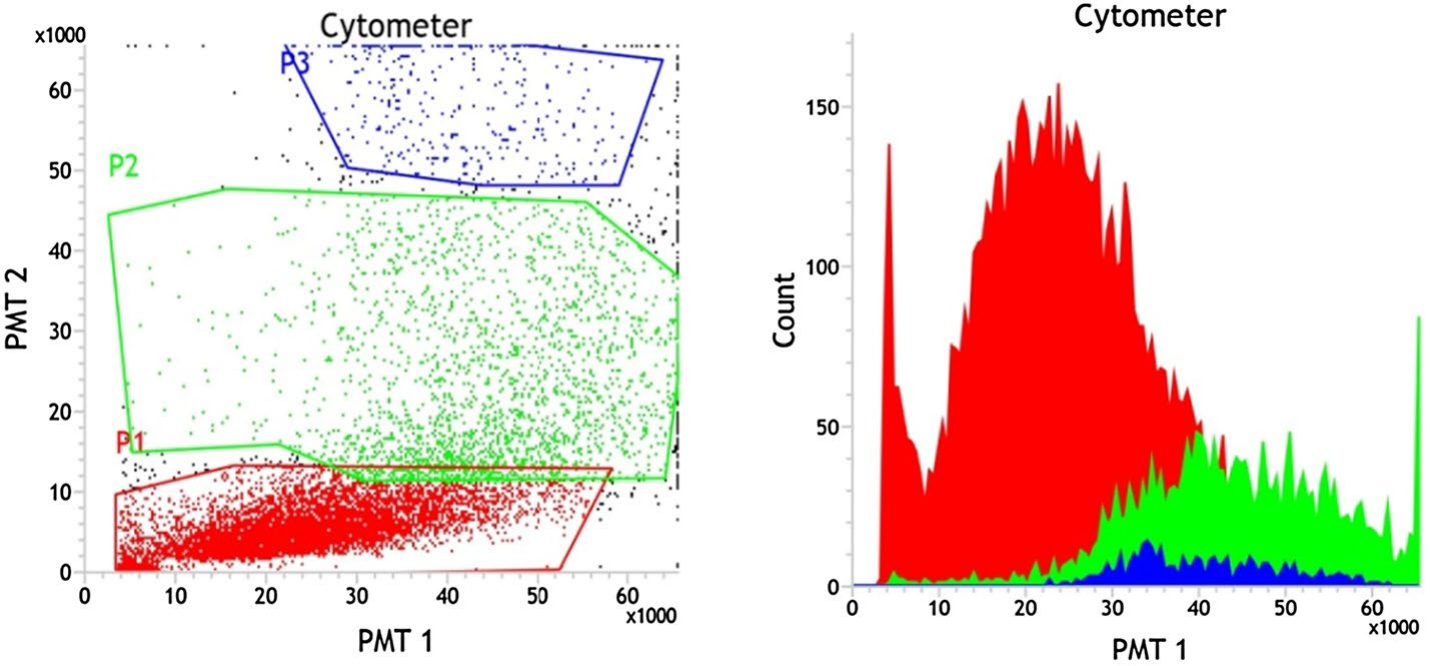
Flow cytometry analysis of hemocyte population in the hemolymph of *Paratelphusa jacquemontii.* Hemolymph (100 µl) collected in 2 ml in iso-osmotic buffer (50 mM Tris, 210 mM NaCl, 5 mM KCl, 2.5 mM MgCl_2_, 100 mg d-glucose, pH 7.5, 480 mOsm) scattered in flowcytogram in size (FSC) and granularity (SSC) as *P1* granular hemocyte, *P2* semigranular hemocyte and *P3* hyaline hemocyte

The cytogram of the unstained hemocytes in the hemolymph of *P*. *jacquemontii* revealed three hemocyte cell populations using FSC and SSC parameters. The cell population dispersed on the cytogram were defined based on the relative size (FSC values) and granularity (SSC values) and the hyaline population with low FSC and SSC constituted 4.20 ± 1.97 %,the semigranular with intermediate FSC and SSC 24.16 ± 4.51 % and granular cells of high SSC constituted 71.81 ± 5.29 % (Fig. 5).

**Fig. 6:**
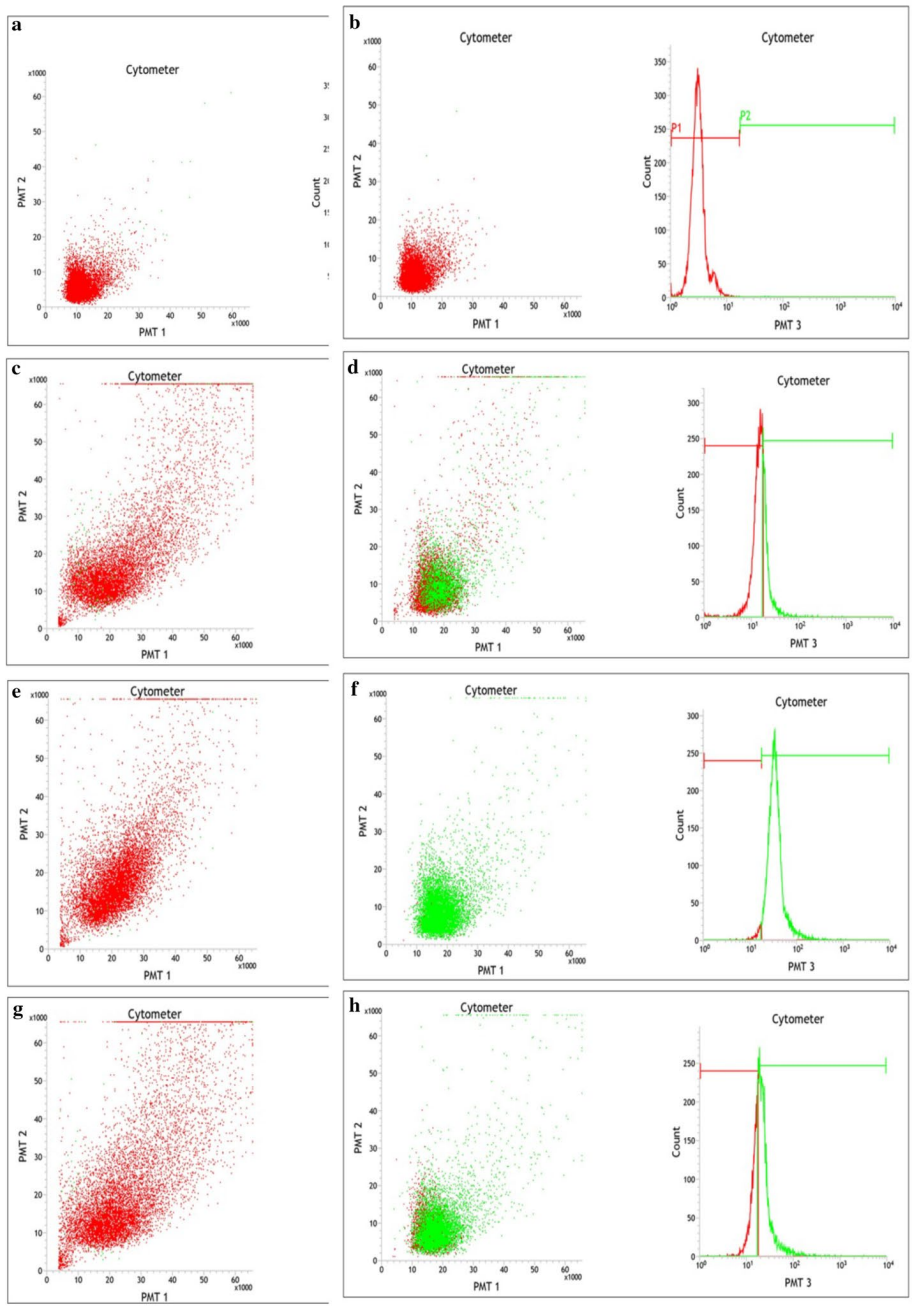
Flow cytometry in vitro phagocytosis analysis of hemocyte population in the hemolymph of *Paratelphusa jacquemontii.* Flow cytogram of in vitro phagocytic activity observed in 100 µl hemocyte suspension in iso-osmotic buffer (Tris 50 mM, NaCl 210 mM, KCl 5 mM, MgCl_2_ 2.5 mM, D-glucose 100 mg, pH 7.5, 480 mOsm) with glutaraldehyde fixed rabbit erythrocyte treated with enzymes and Pjlec. Mean SD: 94.6 2.1 (n 3). Scatter gram was generated by combining forward light scatter (FS) with 7-AAD fluorescence.**a** Untreated rabbit erythrocyte; **b** FITC labeled rabbit erythrocyte; **c** FITC labeled rabbit erythrocyte + hemocytes (2 min); **d** FITC labeled rabbit erythrocyte + hemocytes (30 min); **e** Lectin coated FITC labeled rabbit erythrocyte + hemocytes (2 min); **f** Pjlec lectin coated FITC labeled rabbit erythrocyte + hemocytes (30 min). **g** Trypsin treated FITC labeled rabbit erythrocyte + hemocytes (2 min); **h** Trypsin treated FITC labeled rabbit erythrocyte + hemocytes (30 min)

The flow cytometry analysis of in vivo phagocytosis by hemocytes of *P*. *jacquemontii* was undetected, however in vitro phagocytosis was observed up to 30 min with FITC labeled rabbit erythrocyte (Fig. 6a, b). The phagocytic activity of hemocytes against untreated rabbit erythrocyte was 33.74 % (Fig. 6c, d) and the trypsin treated erythrocyte 68.29 % (Fig. 6e, f), and with lectin coated rabbit erythrocyte 97 % (Fig. 6g, h).

## Discussion

The lectin isolated from the hemolymph of the freshwater crab, *P. jacquemontii* (Pjlec) showed avid binding to erythrocytes comprised of sialic acid on surface glycans, mouse (BALB/c,) horse and rabbit erythrocytes (Denis et al. 2003) For the present study the rabbit erythrocyte that were known to possess N-acetyl,9 neuraminic acid (Neu9Ac) and N-acetyl 5,9 neuraminic acid (Neu5, 9Ac) in surface glycoconjugats (Pfeil et al. 1980) was taken as target cell for the immune responsive function of Pjlec. The *O*-acetyl neuraminic binding of Pjlec was determined as neuraminidase and *O*-glycosidase treated rabbit erythrocyte failed to show HA activity with the lectin. Neuraminidase enzyme from *Clostridium perfringens* appears to cleave sialic acid residues at α-2, 3-, α-2,6- or α-2,8- linkages from the terminal glycoconjugates of the erythrocyte surface (Drzeniek 1967).

However, the influence of proteinase enzymes was negligible that clearly defined the sialic acid binding ability of Pjlec with rabbit erythrocyte. This sialic acid specificity of the lectin was also revealed in vivo studies on induction of lectin activity using rabbit erythrocyte as the target cell. The immunogenic innate immunity response was elicited by observation of enhancement of lectin activity after 2 h of challenge. The sialic acid glycan on the erythrocyte appeared as the molecules required for the reported enhancement of the Pjlec activity as the untreated or proteinase treated erythrocyte were capable of inducing Pjlec activity whereas the asialo erythrocyte was unable to induce lectin activity. This clearly implied Pjlec as an immunogenic protein with specific ability to recognize sialic acid on the non-self molecule. The hemolytic and clearance studies also revealed that the binding of Pjlec facilitated the response revealing the specific response of the lectin to glycan on invading organism was required for an initial recognition and later clearance.

The hemocytes are the effective cellular immune components in crustaceans and in the present study we investigated its immune function of phagocytosis using rabbit erythrocyte as target cell. Based on observations under phase contrast microscope the hemocytes showed phagocytic activity with untreated rabbit erythrocyte and this was enhanced with pre-incubated serum or clarified serum rabbit erythrocyte. The purified lectin Pjlec coated erythrocyte showed a two fold increase in phagocytic activity thereby affirming the role of Pjlec as an opsonin in phagocytosis. However the facilitated phagocytic response of hemocyte was dependent on the lectin Pjlec’s glycan specific binding that was elicited by 27.46 % to that of serum, as elimination of proteins from the serum yielded purified Pjlec lectin. Moreover the proteinase treatment of the erythrocytes enhanced phagocytosis with the removal of inhibitory proteins and exposure of sialic acid on its surface. On the other hand neuraminidase treatment of rabbit erythrocyte failed to be recognized or bind to the hemocytes. These optic observations were supported from the flow cytometry study, where the unstained hemocyte population occurred as three sub populations observed by scattered FSC and SSC in flow cytogram distinguishing the populations of hyaline, semigranular and granular cells. Our study on in vivo phagocytosis with FITC-Annexin labeled rabbit erythrocyte in flow cytometry failed to show response for 30 min after administration of either untreated or Pjlec coated rabbit erythrocyte. Evidently the non self molecule were sequestered from the hemolymph to other organs or cells of immune system probably the fixed hemocytes before being released for phagocytosis by freely circulating hemocytes (Johnson 1987; van de Braak et al. 2002) However by flow cytometry the in vitro phagocytosis analysis by hemocytes were also investigated using the FITC-Annexin labeled native and variously treated rabbit erythrocyte, and revealed phagocytosis in 33.74 % of the hemocyte population and the lectin incubated erythrocyte were engulfed by 97 % of the hemocyte population whereas proteinase treated erythrocytes were ingested by 68.29 % of the hemocytes. Flow cytometry observations of in vitro phagocytic response supported the optic observation of opsonin function of Pjlec in bridging erythrocytes (non-self) to hemocyte by a possible interaction between sialic acid on rabbit erythrocyte and receptors on hemocyte surface. Our findings clearly implicated that the immune response of phagocytosis in *P. jacquemontii* was initiated by recognition involving neuraminic acid binding of Pjlec that acted as an opsonin to interact with hemocyte surface receptors that signal a pathway for a cascade of reactions resulting in ingestion and subsequently elimination of nonself. The protein molecule β-integrin has been reported to attach nonself molecules to hemocyte surface (Wang et al. 2014).The study on in vitro hemolysis affirmed the binding of Pjlec on hemocyte surface to expedite the breakdown of erythrocyte. The observations of in vitro phagocytosis convincingly explained the function of Pjlec in binding the nonself to hemocytes where all the granular and semigranular hemocyte morphotypes appeared to bind to the lectin coated erythrocyte comprising about 94–96 % of the hemocyte population. The hyaline cells that constituted 4–6 % of the hemocyte population failed to phagocytose and appeared as the non-phagocytic proportion of the hemocyte population, indicating that recognition and ultimate ingestion on non self was discriminated at the hemocyte surface receptors (Gargioni and Barracco 1998). The opsonin factor Pjlec had the ability to bind to the phagocytic hemocytes disparated by its surface receptors to recognize, bind and ingest nonself entity and this observation finds support from the findings of (Hose et al. 1990).

The rabbit erythrocyte with neuraminic acid on its cell surface had immunogenic ability to induce lectin activity and it can be suggested that the lectin synthesis was a causal mechanism induced by activation of phagocytosis or vice versa requiring induction of lectin to activate phagocytosis. However Pjlec as a humoral factor showed agglutinin and opsonin function for immune response against the pathogens. The study makes it clear that Pjlec acts as an opsonin that binds specifically to NeuAc and its *O*-acetyl derivatives on the surface of pathogens and binds to the receptors on hemocyte surface to facilitate phagocytosis.

The study also has evolutionary implication, Pjlec a sialic acid binding lectin acts as an opsonin by binding to sialic acid glycan of non-self similar to the surfactant protein A (SP-A) in human that acts by its sialic acid residues as an opsonin in the phagocytosis of influenza A virus by alveolar macrophages (Benne et al. 1997). The recognition of glycan antigen structures by lectin mediated cellular defense mechanism in an innate immune system of the crab apparently has developed into an adaptive response in mammals against infection as multivalent glycoconjugate vaccines have been developed based on the capsular polysaccharides against *Streptococcus pneumoniae*, *Neisseria meningitides* and *Haemophilus influenza* (Zuercher et al. 2006; Buskas et al. 2004).

## Conclusion

The specific recognition of sialic acid on glycan cell surface of non-self in innate immune response of the crab *P.jacquemontii* reflected the specific adaptive response in mammals. The study clearly defined the function of sialic acid specific lectin Pjlec in *P.jacquemontii* as an opsonin in facilitating phagocytosis of non-self indicating the specific recognition of non-self depending on the glycan structure and not the protein on cell surface. Also recognition of the opsonin or Pjlec was confined to the semigranular and granular hemocytes, redefining the specific occurrence of receptor on hemocyte surface. It also implicated the effectiveness of polysaccharide specific vaccines against pathogenic infections. Since the glycoconjugates on cell surface of pathogens are diverse and complex the specific sialic acid binding character of lectin acts as a probe in identification of the binding site of the pathogens. That proves useful for medical and therapeutic research.

## Abbreviation

*Pjlec*: *Paratelphusa jacquemontii* lectin
